# No evidence of tactile distance anisotropy on the belly

**DOI:** 10.1098/rsos.180866

**Published:** 2019-03-13

**Authors:** Matthew R. Longo, Anamaria Lulciuc, Lenka Sotakova

**Affiliations:** Department of Psychological Sciences, Birkbeck, University of London, London, UK

**Keywords:** touch, size perception, tactile distance

## Abstract

The perceived distance between two touches has been found to be larger for pairs of stimuli oriented across the width of the body than along the length of the body, for several body parts. Nevertheless, the magnitude of such biases varies from place to place, suggesting systematically different distortions of tactile space across the body. Several recent studies have investigated perceived tactile distance on the belly as an implicit measure of body perception in clinical conditions including anorexia nervosa and obesity. In this study, we investigated whether there is an anisotropy of perceived tactile distance on the belly in a sample of adult women. Participants made verbal estimates of the perceived distance between pairs of touches oriented either across body width or along body length on the belly and the dorsum of the left hand. Consistent with previous results, a large anisotropy was apparent on the hand, with across stimuli perceived as larger than along stimuli. In contrast, no such bias was apparent on the belly. These results provide further evidence that anisotropies of perceived tactile distance vary systematically across the body and suggest that there is no anisotropy at all on the belly in healthy women.

## 1. Introduction

In his classic studies in the nineteenth century, Weber [1] reported a curious observation that when he moved the two points of a compass across his skin, it felt to him as if the distance between the points increased as he moved them from a region of relatively low sensitivity (such as the forearm), to one of higher sensitivity (such as the palm of the hand). Subsequent studies have replicated this pattern, showing a systematic relation between tactile acuity and perceived tactile distance [2–6]. Analogous perceptual distortions have been described comparing stimuli in different orientations on a single skin surface, with distances feeling larger in some orientations than in others (i.e. an *anisotropy* for tactile distance perception). For example, using a two-alternative forced-choice paradigm, Longo & Haggard [7] found that pairs of touches oriented across the width of the hand dorsum are perceived as approximately 40% farther apart than the same touched oriented along the length of the hand. This effect has been replicated by several subsequent studies [8–17]. Longo and Haggard suggested that both the classic Weber's illusion and anisotropies in perceived tactile distance might result from the geometry of receptive fields (RFs) in somatosensory cortex, which are both smaller on highly sensitive skin surfaces than on less sensitive surfaces [18,19] and, at least on the limbs, tend to be oval-shaped rather than circular, being longer in the proximo-distal limb axis than in the medio-lateral limb axis [20,21].

While the majority of studies investigating anisotropies in tactile distance perception have focused on the hands, a few studies have also reported analogous anisotropies on other body parts, including the thigh [22], the forearm [22,23], the forehead [11,16] and the shin [24]. Intriguingly, in each of these cases the bias is to perceive tactile distances as larger when oriented with the width of the body part than with its length. The consistency of the direction of these effects suggests that overestimation of tactile distances aligned with body width may be a general characteristic of somatosensory organization, potentially connected to overestimation of body width in other tasks [25–31].

There are, however, also hints that tactile distance anisotropies differ in magnitude across the body, and may be absent on some body parts. For example, there is evidence that anisotropies are larger on the forearm than on the hand dorsum [23], larger on the dorsum than on the forehead [11], and smaller on the belly than either the forearm or forehead [32]. Green [22] found clear anisotropies on the hand dorsum and thigh, but did not find such effects on the palm of the hand or on the belly. In the case of the palm, studies have found different results with some studies finding no anisotropy [7,15,22] and others finding an anisotropy qualitatively similar to but smaller than on the dorsum [11,16,23]. The reasons for the difference between these studies are not clear. To our knowledge, anisotropy on the belly has only been assessed in the studies of Green [22] (he calls it the ‘stomach’) and Marks *et al.* [32] (they call it the ‘abdomen’). In Green's study there was a modest trend towards an anisotropy on the belly, which was presumably not statistically significant, though no statistical test of this is reported. In the study of Marks *et al.*, there is no apparent difference as a function of orientation on the belly, though again no statistical test is reported, and a very small sample of six participants was tested. Another study by Spitoni *et al.* [33] also applied stimuli in two orientations on the belly, but compared each to an equivalently oriented stimulus on the sternum, which only allows assessment of the relative anisotropy between those two skin surfaces, not absolute anisotropy on each surface.

Over the past few years, tactile distance perception on the torso has emerged as a common measure in studies investigating body perception in eating disorders and obesity [33–38]. For example, Keizer *et al.* [34,35] have found that patients with anorexia overestimate tactile distances on both the belly and arm compared to control participants. Spitoni *et al.* [33], intriguingly, found that patients with anorexia showed relative overestimation of tactile distances on the belly compared to the sternum only when stimuli were oriented with body width, but not when oriented along the length of the body. However, given that judgements always involved a comparison between the belly and sternum, these data do not provide information about absolute anisotropy on the belly in either patients or controls.

In the present study, we investigated whether there is an anisotropy in tactile distance perception on the belly in a non-clinical sample of women. Participants made verbal estimates of the distance between two simultaneously-presented tactile stimuli oriented either across the width of the belly or along its length. For comparison, we also applied stimuli to the hand dorsum, a region known to have a large anisotropy.

## 2. Method

### 2.1. Participants

Thirty-seven women between 20 and 60 years of age participated. All were right-handed by self-report and reported no tactile abnormalities on the hand or abdomen. On average, participants were 68.4 kg (s.d. = 15.2) in weight, 164.5 cm (s.d. = 7.3) in height, and had a mean BMI of 25.4 (s.d. = 6.1). All participants gave written informed consent before participating. Procedures were approved by the Department of Psychological Sciences ethics committee at Birkbeck, University of London, and were in accordance with the principles of the Declaration of Helsinki.

A weighted average of effect sizes from 15 previous experiments from our laboratory (total *N* = 300) investigating anisotropy for tactile distance on the hand dorsum produced an average Cohen's *d* of 1.56. A power analysis using G*Power 3.1 with alpha of 0.05 and beta of 0.95 suggested a necessary sample of eight participants. Our study is thus well powered to identify a potential anisotropy on the belly. Indeed, if an anisotropy on the belly was even half the size of that on the hand, our study would have more than 95% power to detect it.

### 2.2. Procedures

The stimuli were pairs of wooden sticks embedded in foamboard at different distances, similar to those we have used previously [7,8,11–13,16]. The sticks tapered to a point of approximately 1 mm, but were not sharp. Stimuli were applied to the skin manually by an experimenter for approximately 1 s and with moderate pressure.

To allow access to the abdomen, participants were asked to lie down on an air mattress for the duration of the experiment. They were asked to raise their shirt to provide access to the lower half of their abdomen. During the hand stimulation blocks, they rested their left hand flat on a box to the side of the mattress. Participants were blindfolded throughout the experiment and were tested by a female experimenter.

The experiment was divided into four blocks of 36 trials each. Each block involved stimulation of either the dorsum of the left hand or the abdomen just above the navel. Each block consisted of six repetitions of each combination of two orientations (across, along) and three stimulus distances, in random order. Due to an experimenter error, for 22 participants the final two trials of each block were left off the stimulus sheet, resulting in two random trials from each block not being tested.

On the hand, stimuli in the across orientation were aligned with the medio-lateral limb axis and stimuli in the along orientation were aligned with the proximo-distal limb axis. Stimuli were presented approximately in the centre of the hand dorsum, but were moved slightly from trial-to-trial to avoid repeated stimulation of exactly the same skin location. On the abdomen, stimuli were presented on the belly just above the navel. Stimuli in the across orientation were perpendicular to the body midline, and stimuli in the along orientation were parallel to the body midline.

On the hand, the three actual stimulus distances used were 2, 3 and 4 cm, consistent with previous studies in our laboratory. However, given the lower tactile acuity on the torso than on the hand [39,40], we decided to use a larger set of stimuli on the abdomen (3, 4.5 and 6 cm).

The participant's task on each trial was to estimate the perceived distance (in cm) between the two touches using a verbal response, similar to other studies [8,15,16,41]. Responses were unspeeded and participants were instructed to be as precise with their judgements as possible and to consider using decimal responses (e.g. 3.2 cm rather than just 3 cm). If they were more comfortable using inches they were allowed to do so. They were allowed to give a response of 0 cm if they felt only one touch.

## 3. Results

**Figure 1.**
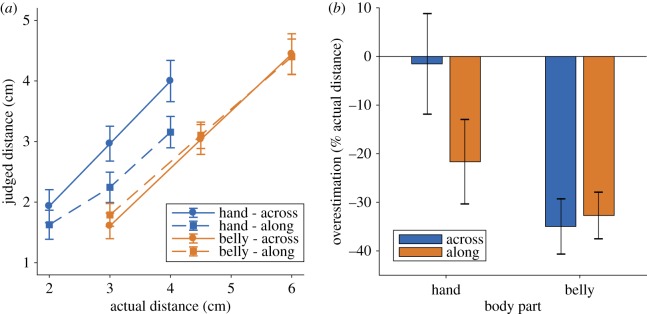
(*a*) Judged distance as a function of actual distance. For the hand, there was a clear anisotropy, with stimuli judged as farther apart when oriented across the hand than along the hand, consistent with previous results. On the belly, in contrast, no such anisotropy was apparent. Error bars are one s.e.m. (*b*) The same data expressed as overestimation as a percentage of actual stimulus size and averaged across the different actual distances. Positive numbers indicate overestimation, while negative numbers indicate underestimation.

The results are shown in figure 1*a*. Judged distance increased monotonically with actual distance in all conditions. An analysis of variance (ANOVA) on the hand showed a significant main effect of actual distance, *F*_1.31,47.15_ = 178.39, *p* < 0.001, $\eta_{p}^{2}=0.83.$ There was also a significant main effect of orientation, *F*_1,36_ = 45.21, *p* < 0.001, $\eta_{p}^{2}=0.56,$ with substantially larger judgements for stimuli oriented with hand width than along hand length, replicating the anisotropy reported previously [7,8,22]. The effect of orientation was modulated by an interaction, *F*_2,72_ = 13.15, *p* < 0.001, $\eta_{p}^{2}=0.27,$ which showed that the bias to judge across stimuli as bigger than along stimuli increased with actual distance, as reported previously [8].

An analogous ANOVA on the belly showed a very different pattern of results. While there was a clear main effect of actual distance, *F*_1.14,41.17_ = 139.89, *p* < 0.001, $\eta_{p}^{2}=0.80,$ there was no main effect of orientation, *F*_1,36_ = 0.31, n.s., $\eta_{p}^{2}=0.01,$ nor an interaction, *F*_2,72_ = 1.41, n.s., $\eta_{p}^{2}=0.04.$ There was thus no evidence for an anisotropy of perceived tactile distance on the belly.

Because different actual stimulus sizes were used on the hand and belly, data from the two body parts cannot be included in a single ANOVA including actual size as a factor. To directly compare the two body parts, we therefore re-expressed each judgement in terms of percentage overestimation of actual distance and collapsed across the three distances used for each body part. These data are shown in figure 1*b*. We then conducted a 2 × 2 ANOVA with body part (hand, belly) and orientation (across, along) as factors. There was a clear main effect of body part, *F*_1,36_ = 15.38, *p* < 0.001, $\eta_{p}^{2}=0.30,$ with greater underestimation on the belly than on the hand. This result is consistent with the classic form of Weber's illusion [1–4], in which perceived distance between two touches is larger on a region of high sensitivity than one of lower sensitivity. There was also a main effect of orientation, *F*_1,36_ = 12.91, *p* < 0.001, $\eta_{p}^{2}=0.5826,$ and an interaction of body part and orientation, *F*_1,36_ = 50.04, *p* < 0.001, $\eta_{p}^{2}=0.58.$

To explore this interaction, we conducted follow-up *t*-tests comparing the two orientations on each body part using Holm–Bonferroni correction for multiple comparisons. On the hand, there was a large bias for judged distance to be larger for stimuli in the ‘across’ orientation than the ‘along’ orientation (−1.53% versus −21.65%), *t*_36_ = 6.29, *p* < 0.0001, *d_z_* = 1.03. In contrast, no such anisotropy was apparent on the belly (−34.98% versus −32.71%), *t*_36_ = −0.85, n.s., *d_z_* = 0.14.

In order to determine whether the lack of a significant anisotropy on the belly provides positive support for the null hypothesis of no effect of orientation, we conducted Bayesian *t*-tests using JASP 0.8.1.1 [42] to compare the two orientations on each body part. Because we had a strong directional prediction that any anisotropy was likely to reflect across stimuli being perceived as larger than along stimuli, we compared that directional alternative hypothesis to the null hypothesis of no difference, using the default parameters in JASP. On the hand, there was decisive evidence in favour of the alternative hypothesis, *BF_+0_* = 110848.35. In contrast, on the belly, there was moderate evidence in favour of the null hypothesis, *BF_0+_* = 9.73.

## 4. Discussion

These results provide evidence against there being an anisotropy in tactile distance perception on the belly in healthy women. Judged distance increased linearly with actual distance, demonstrating the participants were able to perform their task effectively. Nevertheless, judgements were highly similar in the across and along orientations, suggesting that there is no systematic distortion in tactile space on the belly. In contrast, there was a large anisotropy on the dorsum of the hand, consistent with previous results [7,8,11].

Our finding that tactile distance perception on the belly is not anisotropic provides important baseline information for interpreting data from clinical populations [33–38]. We did not explicitly screen our participants for conditions such as eating disorders, and on average our participants (despite having a wide range of BMIs) were slightly overweight. Nevertheless, our results do provide information on anisotropy in an unselected university sample of adult women in the UK. Our results are particularly interesting in the light of the study of Spitoni *et al.* [33], who found that patients with anorexia, but not healthy controls, showed relative overestimation of tactile distances on the belly in comparison to stimuli on the sternum. Combined with the present results, this suggests that anorexia is associated with an anisotropy in tactile distance perception on the belly that does not exist in healthy individuals. Other studies have found that factors such as illusory modification of perceived body size and shape [4,43–45] and tool use [9,10,46,47] alter the perceived size of tactile stimuli. It is thus an intriguing possibility that the orientation specific bias in touch described by Spitoni *et al.* may be an implicit proxy of body image distortions known to characterize patients with eating disorders [48–55], particularly given concerns that such distortions may reflect demand characteristics [56] or attitudinal (rather than perceptual) aspects of body image [57,58].

These results provide additional evidence that anisotropy is not a universal feature of tactile organization. Nevertheless, anisotropy is present on a number of different body parts, including the hand [7,8,11,15], the forearm [22,23,32], the forehead [11,16], the thigh [3,22] and the shin [24]. Moreover, when present, anisotropies in every case reported to date involve overestimation of distances aligned with body width, compared to body length or height. This suggests that anisotropy does not vary idiosyncratically across the body, but that there is a general bias to overestimate body width, a pattern which our data suggest does not hold for the belly. Cholewiak [3] suggested that anisotropy could be related to the organization of dermatomes, which on the limbs are oriented along the proximo-distal limb axes [59–62]. Therefore, a pair of touches oriented across the width of the limb are more likely to fall into different dermatomes, potentially producing expansion of perceived tactile distance as a form of categorical perception effect. Intriguingly, in light of the present results, the organization of dermatomes on the torso is very different as they run the entire width of the torso but are much smaller vertically. Therefore, on the belly, stimuli in the along orientation are much more likely to fall inside different dermatomes than stimuli in the across orientation. Thus, there does appear to be some degree of correspondence between patterns of anisotropy across the body and the organization of dermatomes. However, if anisotropy were determined entirely based on dermatomal organization, there should be a *reversed* anisotropy on the belly, with stimuli in the along orientation perceived as larger, rather than no anisotropy as we found in this study. We have also suggested elsewhere that dermatomal organization may relate to patterns of correlation between the fingers in implicit proprioceptive maps [28].

Recent studies have suggested that perceived tactile distance is expanded across joint boundaries [23,63,64], suggesting that the categorical segmentation of the body into parts affects spatial perception of touch. Another potential form of categorical perception could be based on the body midline, particularly given that tactile inputs from each size of the midline project primarily to the contralateral somatosensory cortex [65]. Recent findings that perceived tactile distance is expanded across the width of the forehead [11,16], for example, could potentially reflect such a categorical perception effect, since in both those studies stimuli in the across orientation involved presenting stimuli on either side of the body midline. Likewise, in the present study stimuli in the across orientation on the belly straddled the body midline. It is noteworthy, therefore, that since we found no anisotropy, these results provide no evidence of a categorical perception effect related to the body midline.

Studies have used a range of methods to investigate tactile distance perception, including two-alternative forced-choice (2AFC) judgements of which of two stimuli is larger [4,7,9–12,43,66], magnitude estimates using either an arbitrary scale [3,22] or (like the present study) a number in cm [8,15,16,41,63], and kinaesthetic estimates made by matching the distance with two fingers [34,35,64]. To our knowledge, no direct comparison between these tasks has been conducted. It is notable, however, that similar results have been obtained in each case. For example, similar tactile anisotropies on the hand dorsum have been reported in studies using 2AFC tasks [7,11,12] and verbal estimates [8,15,16]. Similarly, comparable categorical perception of tactile distance at the wrist has been reported using 2AFC [23], verbal estimates [63] and kinaesthetic estimates [64]. The general similarity of results across these methods is reassuring in suggesting that effects are not due to the task-demands of specific methods.

## Supplementary Material

Raw Data

## Data accessibility

Our data are available as electronic supplementary material.
